# Liposomal CRISPR/Cas9-Mediated Local Genome Editing for Joint Disease in Mucopolysaccharidosis Type I

**DOI:** 10.3390/pharmaceutics18030281

**Published:** 2026-02-24

**Authors:** Hallana Souza Santos, Edina Poletto, Luisa Natalia Pimentel Vera, Mirian Farinon, Francyne Kubaski, Paola Barcelos Carneiro, Willian da Silva Carniel, Roberto Giugliani, Ursula Matte, Helder Ferreira Teixeira, Roselena Silvestri Schuh, Guilherme Baldo

**Affiliations:** 1 Laboratório Células, Tecidos e Genes do Hospital de Clínicas de Porto Alegre, R. Ramiro Barcelos, 2350, Porto Alegre 90035-903, RS, Brazil; hallanasouzas@gmail.com (H.S.S.); edinapoletto@gmail.com (E.P.); luisa.pimentel.vera@gmail.com (L.N.P.V.); pa0labarcellosca@gmail.com (P.B.C.); umatte@hcpa.edu.br (U.M.); gbaldo@hcpa.edu.br (G.B.); 2 Programa de Pós-Graduação em Genética e Biologia Molecular da Universidade Federal do Rio Grande do Sul (UFRGS), Departamento de Genética, Campus do Vale, Av. Bento Gonçalves, 9500, Porto Alegre 91501-970, RS, Brazil; fkubaski@udel.edu (F.K.); rgiugliani@hcpa.edu.br (R.G.); 3 Programa de Pós-Graduação em Ciências Médicas da Universidade Federal do Rio Grande do Sul (UFRGS), Departamento de Medicina, Campus Saúde, Av. Ramiro Barcelos, 2400, Porto Alegre 90035-003, RS, Brazil; mirianfarinon@hotmail.com; 4 Programa de Pós-Graduação em Ciências Farmacêuticas da Universidade Federal do Rio Grande do Sul (UFRGS), Faculdade de Farmácia, Av. Ipiranga, 2752, Porto Alegre 90610-000, RS, Brazil; carnielwillian@gmail.com (W.d.S.C.); helder.teixeira@ufrgs.br (H.F.T.); 5 Programa de Pós-Graduação em Fisiologia da Universidade Federal do Rio Grande do Sul (UFRGS), Instituto de Ciências Básicas da Saúde, R. Sarmento Leite, 500, Porto Alegre 90035-190, RS, Brazil

**Keywords:** CRISPR/Cas9, gene therapy, intra-articular, liposome, microfluidics, mucopolysaccharidosis type I, nonviral vector

## Abstract

**Background/Objectives**: Mucopolysaccharidosis type I (MPS I) is a lysosomal storage disorder caused by α-L-iduronidase (IDUA) deficiency, leading to progressive glycosaminoglycan (GAG) accumulation and severe joint involvement. Gene editing represents a promising alternative to restore localized enzyme production. Therefore, this study aimed to evaluate the feasibility, efficacy, and safety of in situ genome editing through intra-articular administration of a nonviral CRISPR/Cas9 system to increase localized IDUA expression in an MPS I mouse model. **Methods**: Cationic liposomes were formulated to deliver plasmids encoding the CRISPR/Cas9 system targeted to the *ROSA26* locus along with an *IDUA* donor sequence. In vitro assays were performed in fibroblast-like synoviocytes (FLSs) isolated from MPS I mice to assess cytotoxicity, gene editing efficiency, and IDUA activity. In vivo, MPS I mice received intra-articular injections in the knee joints, either as a single dose (short-term study) or monthly for three months (long-term study). IDUA activity, GAG levels, and genome editing efficiency were evaluated in joint tissues, synovial fluid, serum, and major organs. **Results**: Gene-edited FLS showed sustained IDUA activity for up to 30 days with low cytotoxicity. In vivo, intra-articular administration resulted in a significant increase in IDUA activity in joint tissue and synovial fluid without detectable systemic IDUA. Long-term treatment led to persistent joint-localized IDUA activity, significant reductions (>50%) in GAG levels, and detectable genome editing in joint DNA. **Conclusions**: Intra-articular delivery of CRISPR/Cas9 via cationic liposomes enables safe and effective localized genome editing, representing a promising strategy for treating joint manifestations of MPS I.

## 1. Introduction

Mucopolysaccharidoses (MPSs) are a group of inborn errors of metabolism, caused by deficiencies in lysosomal enzymes responsible for the degradation of glycosaminoglycans (GAGs). The accumulation of GAGs affects important cellular processes, such as the trafficking of molecules, endocytosis and autophagy. It also triggers a cascade of responses, altering cellular homeostasis and leading to multisystemic dysfunctions [1,2,3].

Mucopolysaccharidosis type I (MPS I) is a lysosomal storage disorder resulting from deficient or absent activity of the enzyme α-L-iduronidase (IDUA; EC 3.2.1.76), which is essential for the degradation of the glycosaminoglycans (GAGs) heparan sulfate and dermatan sulfate. Impaired IDUA function leads to the progressive accumulation of these substrates within lysosomes, causing multisystemic clinical involvement. A broad range of manifestations, including coarse facial features, organomegaly, developmental delay, and significant skeletal and joint abnormalities, characterizes the disease. The clinical presentation varies according to the residual enzymatic activity determined by specific pathogenic mutations in the *IDUA* gene. This variability gives rise to a spectrum of phenotypes, ranging from the severe form, known as Hurler syndrome, to intermediate Hurler–Scheie syndrome, and the attenuated form, referred to as Scheie syndrome [1,4].

Currently available treatments for MPS I are enzyme replacement therapy (ERT) and hematopoietic stem cell transplantation (HSCT) [1,4]. These options are not completely effective because they are not capable of delivering therapeutical levels of the enzyme to joints, bone, brain, aorta, and the heart valves. Thus, it is necessary to search for new alternative therapies that are efficient in all clinical conditions of the disease [5,6].

Since current treatments do not correct bone or joint conditions [7], persistent orthopedic complications reduce the quality of life of patients due to the progressive characteristic of the disease [1]. In this sense, gene therapy can be an alternative or complementary treatment to traditional therapies [5,6]. 

Gene therapy could enable MPS cells to produce lysosomal enzymes, which could be exported to the extracellular fluid and taken up by neighboring cells via mannose-6-fosfate (M6P) receptors (a mechanism called cross-correction) [8,9]. Due to these post-translational modifications, gene delivery directly to a small number of cells could provide enough enzyme to reduce GAGs accumulation in the tissue, increasing the chances of a successful treatment for MPS I. 

In a previous study [5], our research group demonstrated the feasibility of intra-articular gene therapy in MPS I mice. The results showed high gene expression and IDUA activity in the synovial fluid, although this activity was transient since the plasmid was episomal and did not integrate in the genome.

The CRISPR system (Clustered Regularly Interspaced Short Palindromic Repeats) and its associated protein-9 (Cas9) is the most widely used tool for genome editing nowadays. The platform consists of an endonuclease (usually Cas9) and a guide-RNA. This complex recognizes the target gene sequence, and it cleaves the double-stranded DNA. Adding a donor DNA sequence allows for homologous recombination to insert a new sequence encoding a functional protein [6,8,9].

The administration of liposomes as nonviral carriers of the CRISPR/Cas9 system was performed in previous studies. Increased IDUA activity and reduction in lysosomal mass was observed in human fibroblasts of MPS I patients in vitro. In addition, the intravenous administration of the complex to newborn MPS I mice in vivo increased IDUA activity and reduced GAGs accumulation in the main organs [6]. However, the delivery of sufficient enzyme to joints to prevent or improve the symptoms of the impaired mobility of MPS I animals and patients remains a challenge to be addressed.

Thus, the intra-articular injection in the main joints would be an alternative, as the exogenous gene would be delivered directly to the joint cavity, potentially improving the efficiency of the therapy in this organ. In addition, the localized administration promotes a safe approach because the joints are closed cavities and non-target organs would not be affected [10]. The idea of using cationic liposomes carrying plasmids of the CRISPR/Cas9 system would be a possible answer to this problem. The liposomal complexes can diffuse and cross the synovial membrane due to the fenestrated sieving effect existing in the endothelium of sub-synovial capillaries. Gene products would be able to cross the synovial membrane, and this would lead to sustained protein synthesis in the joints [11,12].

In this context, this study aimed to evaluate the efficiency of genome editing in situ through the intra-articular administration of cationic liposomes carrying plasmids encoding for the CRISPR/Cas9 system plus a donor vector to insert the *Idua* gene in vivo, to increase localized production of IDUA in an animal model of MPS I.

## 2. Materials and Methods

### 2.1. Vectors 

The Precision X CRISPR/Cas9 SmartNuclease™ system (System Biosciences, Palo Alto, CA, USA) was used for both in vitro and in vivo experiments. The target sequence to be cleaved by the Cas9, 5′ ggattctcccaggcccaggg 3′, was selected at the ROSA26 locus of the mouse genome and was cloned in the vector. A vector containing the IDUA cDNA (System Biosciences, Palo Alto, USA) was used for homologous recombination. The construct contains the mouse IDUA cDNA sequence regulated by an EF1-alpha promoter and two homologous regions (approximately 1 Kb each) to the ROSA26 locus of mice, in the region that Cas9 recognizes and cleaves. The plasmid was transformed into Escherichia coli Top 10 (Life Technologies, Carlsbad, CA, USA) using standard procedures and isolated using Maxi Prep™ High Purity columns (Life Technologies, USA) according to the manufacturer’s instructions. The extracted plasmid DNA was then sequenced by the Sanger method to verify the correct orientation of the insert.

### 2.2. Liposomal Formulations

Liposomes (LPs) were produced according to Schuh et al. [12] and were composed of 0.56% (*w*/*w*) phospholipid 1,2-dioleoyl-sn-glycero-3-phosphoethanolamine (DOPE) (Lipoid, Germany), 0.56% (*w*/*w*) 1,2-dioleoyl-sn-glycero-3-trimethylammonium propane (DOTAP) (Lipoid, Ludwigshafen, Germany), and 0.285% (*w*/*w*) 1,2-distearoyl-sn-glycero-3-phosphoethanolamine-N-[amino (polyethylene glycol)-2000] (DSPE-PEG) (Lipoid, Ludwigshafen, Germany). The liposomal formulations were prepared by microfluidization (LV-1 equipment, Microfluidics, Vancouver, BC, Canada). Complexes were obtained by the addition of previously established amounts of DNA at room temperature for 30 min to the liposomal formulation. The Liposome was incubated with CRISPR/Cas9 (C) and IDUA donor (D) plasmid for complex formation LP+C+D or incubated only with IDUA donor for the formation of the complex LP+D.

### 2.3. Physicochemical Properties of the Formulations

Mean droplet size, polydispersity index (P.I.), and zeta potential (ζ-potential) of the formulations and complexes were determined by photon correlation spectroscopy (PCS) at 90° and electrophoretic mobility measurements (3000HS Zetasizer, Malvern Instruments, Malvern, UK). The samples were diluted in water or 1 mM NaCl.

### 2.4. In Vitro and In Vivo Assays

#### 2.4.1. Animals

In total, two-month-old C57BL/6 *Idua* −/− mice (MPS I) (*n* = 30) and normal Idua +/+ mice (*n* = 14) were used for in vitro and in vivo experiments. *Idua* −/− mice were genotyped by PCR reaction [13]. Animals were gently donated by Dr. Elizabeth Neufeld (UCLA, Los Angeles, CA, USA) and were kept at the Animal Experimental Unit of Hospital de Clínicas de Porto Alegre. The experiments followed the norms of adequacy to the current guidelines foreseen in Law 11794/08 and the normative resolutions number 30 (Use of Animals for Scientific and Didactic purposes) and 13 (CONCEA Euthanasia Practice Guidelines). The authors “Institutional Review Board” approved all experiments (CEUA/HCPA 2019-0122, approved on 6 April 2019). 

#### 2.4.2. Isolation and Culture of Fibroblast-like Synoviocytes (FLSs)

The FLSs were isolated and cultured as previously described [14]. Four animals per group (*Idua* −/− and normal *Idua* +/+) were used to collect the cells. The joints were collected and incubated with collagenase I (1 mg/mL) (Sigma-Aldrich, St. Louis, MO, USA) for 1 h at 37 °C. The supernatant was collected and centrifuged at 240× *g* for 10 min and the pellet was resuspended in Dulbecco’s modified Eagle’s medium-high glucose (DMEM-HG) (Gibco—Thermo Fischer Scientific, Waltham, MA, USA), supplemented with 15% fetal bovine serum (FBS) (Gibco/Thermo Fisher Scientific, Waltham, MA, USA), 1% penicillin–streptomycin (Gibco/Thermo Fisher Scientific, Waltham, MA, USA), and transferred to a 96-well plate for culture at 37 °C and 5% CO_2_ atmosphere. The culture was monitored day to day until 70–80% of confluence, when cells were released with trypsin-EDTA (Gibco/Thermo Fisher Scientific, Waltham, MA, USA) and transferred to a 6-well plate until 70–80% of confluence, and posteriorly transferred to a culture flask.

#### 2.4.3. FLS Growth Curve

To determine the growth pattern of the FLS from MPS I and normal mice, the FLSs at passage 5 were seeded in a 6-well plate at a density of 1 × 10^4^ cells/well at 37 °C in 5% CO_2_. At 24, 48, and 72 h cells were detached with trypsin-EDTA (Gibco/Thermo Fisher Scientific, Waltham, MA, USA) and counted using the Neubauer chamber using Trypan blue.

#### 2.4.4. FLS Viability Assay

Vector toxicity was analyzed by assessing FLS viability using MTT assay [15]. Cells were plated in 96-well plates at a density of 1 × 10^4^ cells/well. FLS were allowed to adhere over 24 h and then cultured in the presence or absence of 10 µL LP or 10 μL LP+C+D complexes containing 1.83 µg of each plasmid for 48 h at 37 °C in 5% CO_2_. In the last 4 h, cells were incubated with MTT and the absorbance of each well measured at 570 nm in a spectrophotometer.

#### 2.4.5. In Vitro Gene Editing

The FLS from MPS I mice at passages 2–5 were seeded at 1 × 10^4^ cells/well in a 12-well plate, cultivated in DMEM-HG containing 10% FBS, 1% ampicillin/streptomycin and maintained in a humidified CO_2_ incubator at 37 °C until 50–60% of confluence. Gene transfer was performed by incubating cells for 24 h in serum-free DMEM-HG with 10 μL of LP+C+D or LP+D complexes containing 1.83 μg of each plasmid or complexed with Lipofectamine (LF) 3000^®^ (LF+D+C and LF+D; according to the manufacturer’s instructions). After incubation, 10% FBS was added. Cells were maintained in culture and were collected at 3, 7, 15, and 30 days after transfection. FLS MPS I untreated controls and FLS from normal (wild type) mice were cultured under the same conditions.

#### 2.4.6. Pilot (Short-Term) Study

MPS I mice were treated with one intra-articular injection of 10 µL containing the complex LP+C+D (*n* = 3) or phosphate-buffered saline (PBS) (*n* = 3). Injection into both the tibiofemoral joints was performed after sedation. After 7 days, the animals were euthanized by cervical dislocation under anesthesia. Whole blood was collected and centrifuged to obtain the serum. The articular cavities were washed with 10 µL (PBS) in a final volume of 70 µL. The joints, synovial fluid, serum, liver, and kidneys were isolated and flash-frozen in liquid nitrogen. The material collected was used for biochemical analyses. Knee joints were pulverized using a mortar and pestle filled with liquid nitrogen [16]. 

#### 2.4.7. Extended (Long-Term) Study

MPS I mice were treated with one intra-articular injection of 10 µL containing the complex LP+C+D (*n* = 8), LP+D (*n* = 8) and compared to untreated mice (*n* = 7). Injection into the tibiofemoral joints was performed after sedation, once a month, for 3 months, between the second and fourth months of life. At eight months, mice were euthanized by cervical dislocation under anesthesia. Whole blood was collected and centrifuged to obtain the serum. The articular cavities were washed with 10 µL aliquots of phosphate-buffered saline (PBS) in a final volume of 70 µL. As described in the previous section, the same organs and fluids were collected. 

#### 2.4.8. IDUA Activity

Assessment of IDUA activity was performed in FLS four times (3, 7, 15, and 30 days) after transfection, in body fluids and in tissues. For in vitro experiments, cells were released with trypsin, centrifuged for complete removal of the culture medium, and suspended in purified water. After vortexing cells, pellet was centrifuged and the 20 µL of supernatant was used to assess IDUA activity and protein. Similar procedure was used for serum and synovial fluid. For tissues, 20 mg was homogenized with purified water using Ultraturrax^®^ (Ika, Campinas, Brazil) equipment, followed by centrifugation, and the supernatant was used to assess IDUA activity and protein levels. These samples were incubated with the 4-methyl-umbelliferyl alpha-L-iduronidase substrate (Glycosynth, Warrington, UK), at 37 °C for 1 h [13]. The cleavage of the substrate generates a fluorescent compound that could be measured with 365 nm excitation and 450 nm emission filters in a fluorescence spectrophotometer (SpectraMax M2, Molecular Devices, San Jose, CA, USA). The protein content was quantified using the method described by Lowry and coworkers [17]. Results were calculated as nmol/h/mL in serum and synovial fluid, or as nmol/h/mg protein in FLS and tissues, and are shown as a percentage of the activity obtained in normal mice. FLS cells, tissues, serum, and synovial fluid from treated animals were compared to untreated MPS I mice for statistical comparison.

#### 2.4.9. GAG Levels

Specific GAG levels were assessed by tandem mass spectrometry from synovial fluid, joint, serum, liver, and kidney. Ten microliters of synovial fluid was used. Tissue GAGs were extracted after acetone precipitation. Dermatan sulfate (DS), heparan sulfate with O- or N-sulfation (HS–OS and HS–NS) disaccharides were obtained through digestion of samples with chondroitinase B, heparitinase, and keratanase II, followed by quantification through liquid chromatography tandem mass spectrometry (LC/MS/MS) as previously described [18].

#### 2.4.10. Efficiency of Gene Editing in the Joint 

To estimate the efficiency of gene editing, quantification of *IDUA* gene present in the donor sequence was performed by real-time PCR, as previously described [19]. Genomic DNA was extracted from olfactory bulb, brain frontal cortex, brain total cortex, lung, and heart tissues with the EasyDNA gDNA Purification Kit (Thermo Fisher Scientific, Waltham, MA, USA) according to manufacturer’s instructions and quantified with a NanoDrop 1000 spectrophotometer (Thermo Fischer Scientific, Waltham, MA, USA). The donor plasmid, which contains a single copy of the *IDUA* sequence, was used as a standard. The number of copies per nanogram of plasmid was calculated with the following formula:

$$number\;of\;copies\;=\;\frac{\left(amount\;in\;ng\;\times \;6.02\;\times \;10^{23}\right)}{\left(lenght\;in\;bp\;\times \;1\;\times \;10^{9}\times \;650\right)}$$

Reactions were carried out in a StepOne Real-Time PCR System (Thermo Fisher Scientific, Waltham, MA, USA) with 5 μL of master mix SybrGreen, 0.1 μL of each primer (0.1 mM final concentration), 1.8 μL of nuclease free water, and 60 ng of DNA in a final volume of 10 μL. The following primers were used: forward 5′-CAA GAC CTG CCT GAA ACC GA-3′ and reverse 5′-ATT GAC CGA TTC CTT GCG GT-3′, a flanking region of *ROSA26* and a flanking region in the *IDUA* transgene to verify transgene insertion. All reactions were performed in standard conditions at an annealing temperature of 60 °C. A standard curve of serial dilutions of the donor plasmid in triplicate was included in each run (1:4 serial dilutions from 36,000 to 140 copies of *IDUA* gene). Samples were considered as containing the transgene if amplification occurred before the Ct30 (cycle to threshold). Copy number in tissues was calculated as previously described. The correction efficiency was expressed as the percentage of transgene *IDUA* copy number found in each tissue analyzed relative to the copy number of mouse genomic DNA in the harvested tissues [20].

#### 2.4.11. Statistical Analyzes 

The GraphPad software (v6) was used for statistical analysis. The group differences were analyzed by Student’s T-test or one-way ANOVA, with Tukey as post hoc, as indicated. For the parameters that presented non-parametric data, the Mann–Whitney test was used. Differences were considered statistically significant at * *p* < 0.05, ** *p* < 0.01, and *** *p* < 0.001.

## 3. Results

### 3.1. Physicochemical Properties 

The physicochemical properties of the formulations used in the experiments are listed in Table 1. The mean droplet size of the blank formulation was approximately 90 nm and approximately 110 nm after complexing with the DNA plasmids. Regarding the polydispersity index (P.I.), it was 0.26 for liposome (LP), 0.16 for liposome associated with CRISPR/Cas9 and IDUA donor plasmids (LP+C+D) and 0.19 for LP+D. The formulations demonstrated positive zeta potential (ζ-potential), and this potential was significantly lower after complexing with the DNA plasmids.

### 3.2. Edited Fibroblast-like Synoviocytes Produce IDUA In Vitro

**Figure 1:**
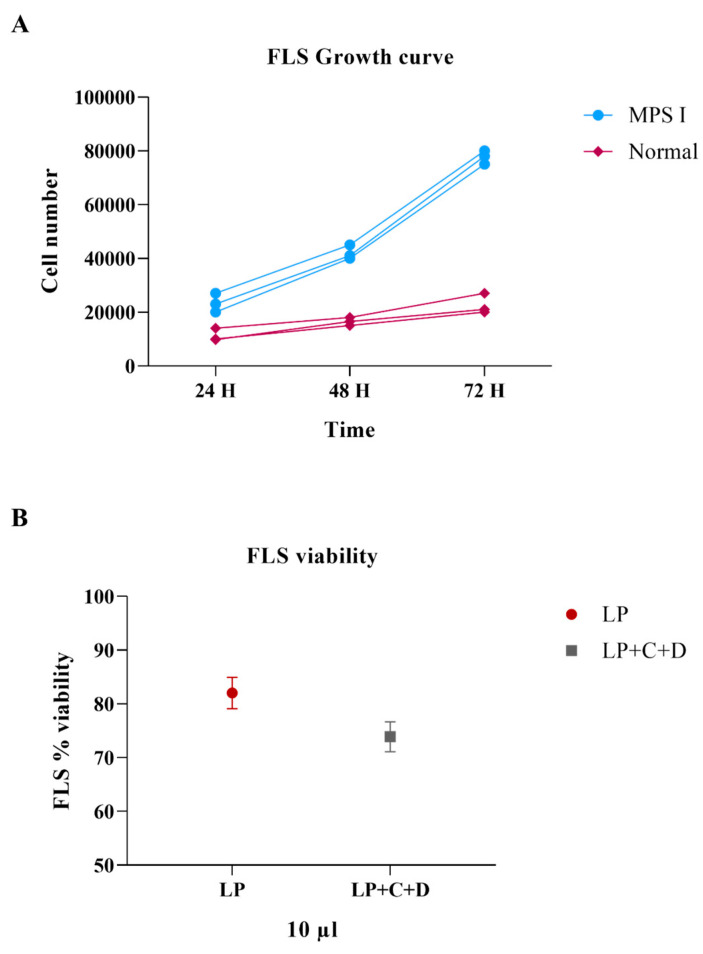
In vitro assays. (**A**) Fibroblast like synoviocytes (FLS) growth curve at 24, 48, and 72 h. FLS from MPS I (blue circles) and normal (pink) mice. (**B**) FLS viability assessed through MTT assay after incubation with 10 µL of Liposome (LP) (wine circles) or 10 µL Liposome+CRISPR/Cas9+Donor IDUA complexes (LP+C+D) (dark grey squares).

Fibroblast-like synoviocytes (FLSs) from the knee joint were obtained and cultivated to perform in vitro tests. The growth curve experiments showed hyperproliferation of MPS I FLS compared to cells from wild type (normal) mice (Figure 1A). FLS viability assay showed that the medium supplementation with 10 µL of Liposome (LP) or Liposome+CRISPR/Cas9+Donor IDUA (LP+C+D) complexes led to high cell viability, higher than 70%. The two treatments showed no statistical difference (*p* > 0.05) (Figure 1B).

**Figure 2:**
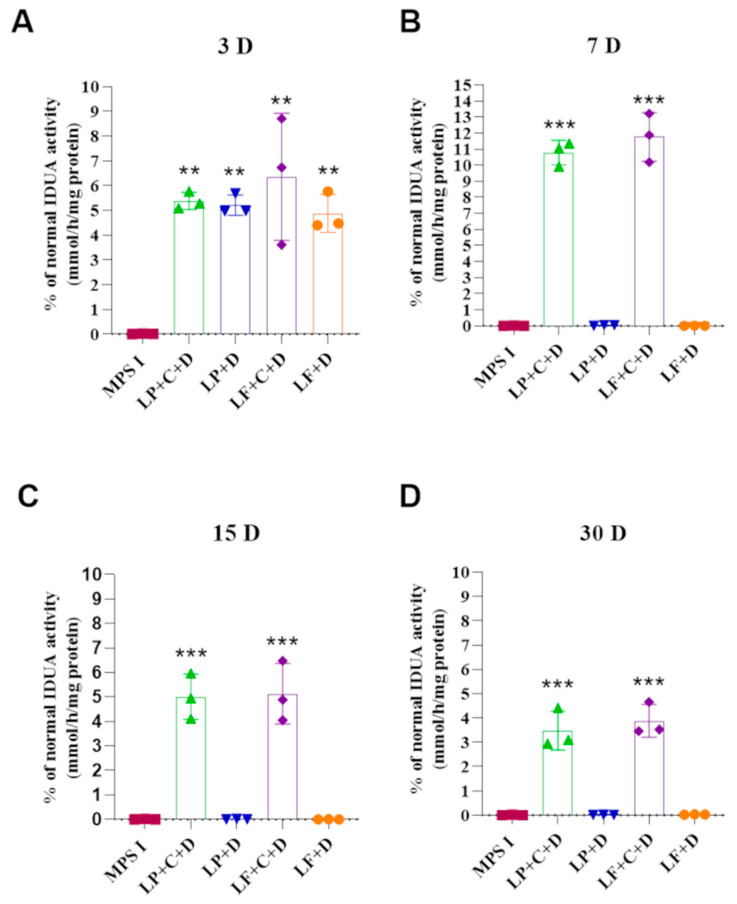
Enzyme activity after 3–30 days of in vitro gene editing. Five groups had their FLS intracellular IDUA assessed: untreated MPS I (MPS I), MPS I FLS treated with our liposome containing either the 2 plasmids for gene editing (LP+C+D) or containing only the donor vector (LP+D) and MPS I FLS treated with the commercially available Lipofectamine3000® containing either the 2 plasmids for gene editing (LF+C+D) or containing only the donor vector (LF+D). At times 3 (**A**), 7 (**B**), 15 (**C**) or 30 (**D**) days. Results are shown as percentage of wild type (normal) cells and represent mean ± standard deviation. Statistical analysis was performed using one-way ANOVA and Tukey’s post hoc test, ** *p* < 0.01, and *** *p* < 0.001 indicated difference from MPS I cells.

IDUA activity in FLS was assessed at 3, 7, 15 and 30 days after incubation with the complexes between the plasmids and lipofectamine or liposome (Figure 2). The treatment with the commercially available Lipofectamine+CRISPR/Cas9+donor IDUA (LF+C+D) or with our vector (LP+C+D) led to a significant increase in IDUA activity at all times (*p* < 0.01 and *p* < 0.001). This increase was sustained for up to 30 days, the last time point analyzed. The treatment with LF+C+D reached an average of 11.8% (±1.5) of IDUA activity found in wild type (normal) FLS, while the treatment with LP+C+D reached an average of 10.8% (±0.8). Both treatments were not significantly different from each other (*p* > 0.05). FLS incubated with the transfection reagents plus only the donor plasmid (without the vector with the Cas9 gene and the gRNA) showed increased IDUA activity only at 3 days. IDUA activity reached an average of 5.3% (±0.5) for Liposome+donorIDUA (LP+D) (*p* < 0.05) and 5.1% (±1.0) for Lipofectamine+donorIDUA (LF+D) (*p* < 0.05). The MPS I FLS produced virtually undetectable levels of IDUA, at 0.004% (±0.005) of normal cells (0.26 ± 0.30 nmol/h/mg).

### 3.3. IDUA Activity Is Increased and Localized in Joint of MPSI Mice After In Situ Gene Therapy

**Figure 3:**
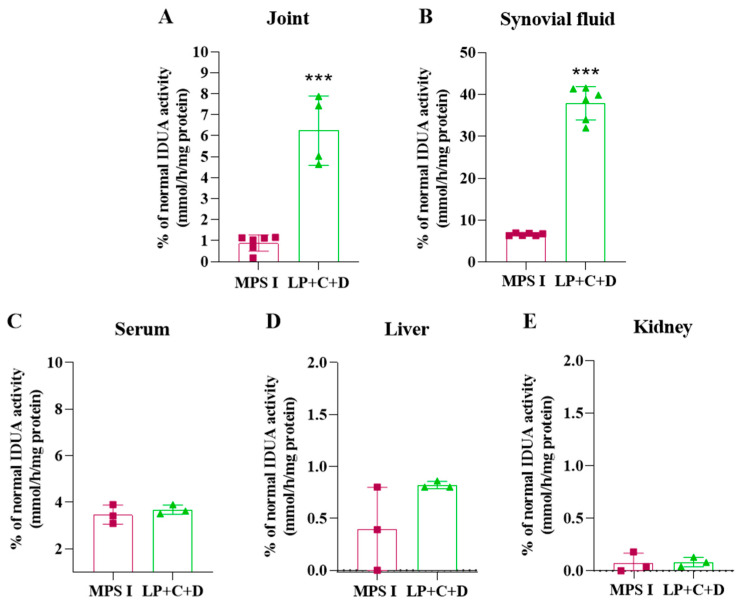
IDUA activity 7 days after a single intra-articular injection of Liposome+CRISPR+donor IDUA (LP+C+D) in MPS I mice (*n* = 3 per group, injecting both joints). IDUA activity in the joint (**A**), synovial fluid (**B**), serum (**C**), Liver (**D**) kidney (**E**), of untreated (fuchsia bars) and treated MPS I mice (green bars). Results represent the mean ± standard deviation. Student’s *t*-test, *** *p* < 0.001.

To evaluate short-term effects of our treatment, mice were injected with a single dose of the gene editing complexes, and seven days after intra-articular injection of Liposome+CRISPR/Cas9+donor IDUA (LP+C+D) in MPS I mice, IDUA enzyme activity was assessed in joints, synovial fluid, serum, liver, and kidneys (Figure 3).

Treated animals showed 6.2% (±0.2) of normal IDUA activity in the joint tissues, representing a significant increase in IDUA activity compared to untreated mice (*p* < 0.001). In the synovial fluid, treated animals showed 38.5% (±2.5) of normal IDUA activity, significantly higher than the untreated group (*p* < 0.001). The enzyme was not significantly increased in serum, liver, or kidney (*p* > 0.05), suggesting that there is little or no shedding of the vector to the circulation.

**Figure 4:**
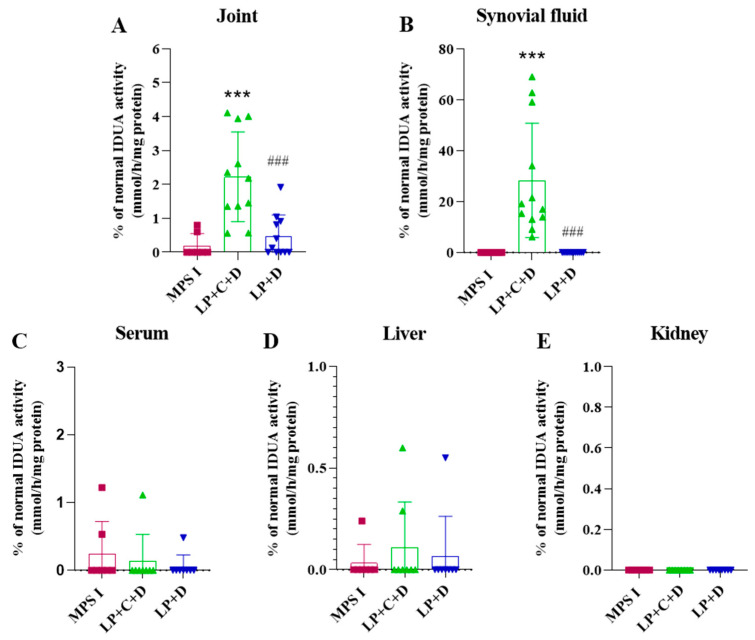
IDUA activity at 8 months, after three intra-articular injections of Liposome+CRISPR +Donor IDUA vectors (LP+C+D), Liposome+Donor IDUA vectors (LP+D) in MPS I mice, compared to untreated animals. IDUA activity in joint (**A**), synovial fluid (**B**), serum(**C**), Liver (**D**), kidney (**E**), and serum of untreated and treated MPS I mice. Results represent the mean ± standard deviation. MPS I treated with LP+C+D and LP+D was compared untreated MPS I group, one-way ANOVA and Tukey’s post hoc test, *** *p* < 0.001. LP+C+D was compared with LP+D (one-way ANOVA and Tukey’s post hoc test), ### *p* < 0.001.

We then sought to evaluate the effects of long-term treatment of our gene editing product. Mice received three monthly administrations, from 2–4 months of life, and were euthanized at 8 months of age. At the endpoint, IDUA activity was assessed in joints, synovial fluid, serum, liver, and kidneys of mice (Figure 4). Additionally, GAG levels and quantification of gene editing efficiency were performed in the joint.

The treatment significantly increased IDUA activity in the joints (*p* < 0.001). Animals treated with the whole vector Liposome+CRISPR+donor IDUA (LP+C+D) showed 2.2% (±1.2) of normal IDUA activity in the joint tissues (*p* < 0.001), while animals treated only with the donor sequence Liposome + donor IDUA (LP+D) and the untreated animals showed low levels of IDUA activity 0.5% (±0.6) and 0.20% (±0.3) respectively. The increase in the group treated with the complete vector was statistically significant compared to the group treated with the donor sequence only (*p* < 0.01), indicating that the editing indeed happened. The treatment significantly increased IDUA activity in synovial fluid as well (*p* < 0.05). Treated animals with LP+C+D showed 25.2% (±20.6) of the normal IDUA activity in the synovial fluid (*p* < 0.001), while the LP+D and the untreated animals showed undetectable levels of IDUA activity.

**Figure 5:**
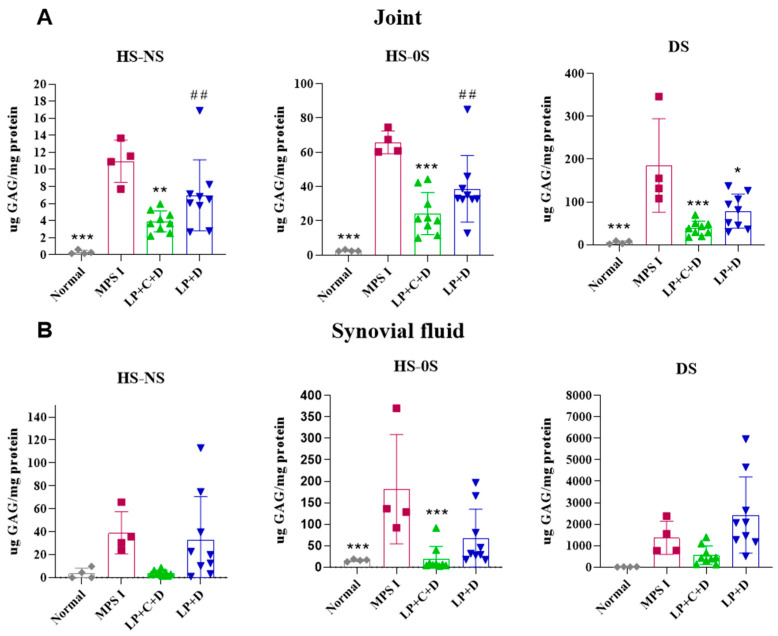
Specific GAG (DS, HS–OS, and HS–NS) levels in joint (**A**) and synovial fluid (**B**) at 8 months, after three intra-articular injections of Liposome+CRISPR+Donor IDUA (LP+C+D) or only Liposome+Donor IDUA (LP+D) in MPS I mice, using tandem mass spectrometry. Normal group formed by untreated C57BL6 Idua +/+ mice. Results represent the mean ± standard deviation. MPS I mice treated with LP+C+D and LP+D were compared to the untreated MPS I group (one-way ANOVA and Tukey’s post hoc test). * *p* < 0.05, ** *p* < 0.01, and *** *p* < 0.001. MPS I mice treated with LP+C+D and LP+D were compared to the normal group (one-way ANOVA and Tukey’s post hoc test). ## *p* < 0.01.

Specific GAG (HS–NS, HS–0S and DS) levels were determined in joints and synovial fluid using tandem mass spectrometry at 8 months (Figure 5). DS, HS–OS, and HS–NS levels in the treated group were reduced by over 50% in the joint (*p* < 0.01). 

In the synovial fluid, HS–NS and HS–0S were significantly reduced (*p* < 0.05). DSs also were also dramatically reduced, but these reductions did not reach statistical significance (*p* > 0.05) possibly due to the variance observed in this assay. LP+D was slightly effective in reducing GAGs, although only DS from joint samples was significantly different from the MPS I group.

**Figure 6:**
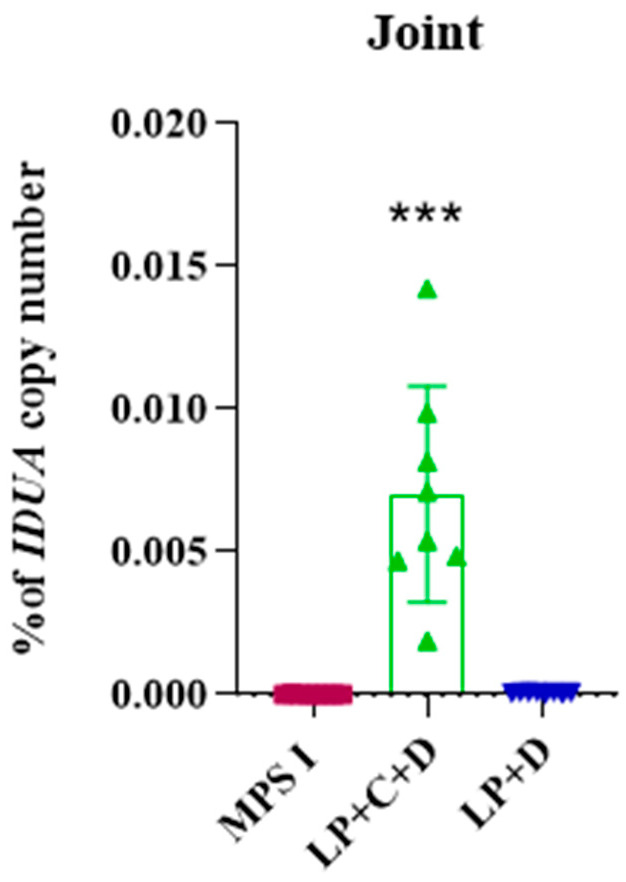
Gene editing efficiency. Gene editing efficiency in treated mice was expressed as a percentage of transgene (Idua) copy number found in the joint from treated mice. Untreated MPS I mice (fuchsia bars), MPS I treated with Liposome+CRISPR+donor IDUA (LP+C+D) (green bars), and MPS I treated with liposome+Donor IDUA (LP+D) (blue bars). Results represent the mean ± standard deviation. Groups compared to untreated MPS I group (one-way ANOVA and Tukey’s post hoc test) *** *p* < 0.001.

The percentage of gene editing was investigated at the DNA level in mice treated with the complete gene editing system (LP+C+D) and with the donor IDUA (LP+D). Absolute quantitative PCR showed 0.007% IDUA copy number (*p* < 0.001) in the joint of treated mice, while no amplification was observed in samples from the other groups (Figure 6).

## 4. Discussion

In this study, we evaluated the efficiency of genome editing in situ through the intra-articular administration of cationic liposomes carrying plasmids encoding for the CRISPR/Cas9 system plus a donor vector to correct *Idua* gene (in vivo) and fibroblast-like synoviocytes (in vitro), to increase localized production of IDUA in an animal model of MPS I. 

The physicochemical results of the formulations used in the study showed that the droplet size obtained and P.I. were expected and appropriate for intra-articular administration. The positive ζ potentials were due to the presence of the cationic lipid DOTAP in the formulations, which is highly essential for the stable complexation with negatively charged nucleic acids [6].

Interestingly, the growth curve experiments showed hyperproliferation of MPS I FLS compared to normal cells. We believe this is due to the hyperplasia observed in the synovial membrane from animals and patients with MPS. The accumulation of GAGs in synovial cells causes increase levels of pro-inflammatory cytokines as tumor necrosis factor (TNF)-alpha, interleukin (IL) and growth factor (TGF-b), which may cause proliferation of these cells [3]. Accumulation of GAGs within lysosomes disrupts normal cellular homeostasis and can trigger chronic inflammatory signaling in the synovial membrane. This inflammatory microenvironment, characterized by cytokine release and macrophage infiltration, promotes synovial cell activation and hyperplasia. The expanded and metabolically active synovial cell population exhibits increased lysosomal biogenesis and turnover as a compensatory response to substrate storage. Consequently, elevated lysosomal enzyme synthesis and secretion may occur, leading to higher detectable enzyme activity in the synovial fluid. Thus, increased lysosomal enzyme levels in synovial fluid may reflect both enhanced production by hyperplastic synovial cells and leakage from stressed or damaged cells within the inflamed joint environment [3]. The FLS viability assay showed high cell viability. These results showed that the LP presented low cytotoxicity, appropriate for gene editing studies [21].

The FLS incubation with the complex LF+C+D and LP+C+D showed increased IDUA activity at all times assessed, while the untreated FLS MPS I showed undetectable levels of IDUA. As expected, FLS incubated with the transfection reagents plus only the donor plasmid, such as LP+D and LF+D, showed increased IDUA activity only at 3 days, which can be explained as the IDUA donor plasmid is expressed, but not incorporated into the genome [12]. Furthermore, the higher IDUA expression observed at Day 7 compared to Day 3 can be explained by the transition from transient to stable transgene expression. At early time points, IDUA activity likely reflects a combination of episomal donor DNA and ongoing homologous recombination events. However, HDR-mediated integration at the *ROSA26* locus is not immediate and may continue for several days following Cas9 cleavage. By Day 7, episomal vectors are expected to be largely lost, while successfully integrated copies become chromatinized and transcriptionally stabilized within the permissive *ROSA26* locus, resulting in more consistent and potentially higher per-cell expression. Additionally, transient transfection-associated stress at early time points may temporarily limit transcriptional output, with recovery over time further contributing to increased expression. Together, these factors provide a plausible explanation for the enhanced IDUA levels observed at Day 7 despite the theoretical limitation of two integration events per cell. 

At the other time points, cells transfected with donor vector only were not different from untreated MPS I, demonstrating that there is no sustained increase in IDUA activity when only the donor plasmid is transfected. These results evidence that the enzyme was being produced in significant amounts when the CRISPR/Cas9 system is provided for FLS and, furthermore, our vector produces similar results to other commercially available liposomes (Lipofectamine3000^®^).

We theorize that the higher IDUA expression observed at Day 7 compared to Day 3 can be explained by the transition from transient to stable transgene expression. At early time points, IDUA activity likely reflects a combination of episomal donor DNA and ongoing homologous recombination events. However, HDR-mediated integration at the ROSA26 locus is not immediate and may continue for several days following Cas9 cleavage. By Day 7, episomal vectors are expected to be largely lost, while successfully integrated copies become chromatinized and transcriptionally stabilized within the permissive ROSA26 locus, resulting in more consistent and potentially higher per-cell expression. Additionally, transient transfection-associated stress at early time points may temporarily limit transcriptional output, with recovery over time further contributing to increased expression. Together, these factors provide a plausible explanation for the enhanced IDUA levels observed at Day 7 despite the theoretical limitation of two integration events per cell. 

Our short-term study in vivo revealed that the intra-articular injection of LP+C+D in MPS I animals increased IDUA activity in the joint and synovial fluid without increase in serum, liver, and kidney as observed previously [5]. These results suggest that the administration of the CRISPR/Cas9 system in the joints promotes gene editing only in situ, without the shedding of the vector to serum or other organs at significant levels. This is particularly important for safety reasons and opens up the possibility to use the same vector to treat other joint conditions as well. 

We then performed the extended (long-term) treatment with our vector, which consisted of three doses of intra-articular injection. The animals treated with LP+C+D showed increased IDUA activity in the joint, while the animals treated with LP+D and the untreated animals showed similarly low levels of IDUA activity, as expected. Together with the qPCR data confirming in the treated mice, these results indicate that the editing indeed happened. It is worth highlighting that chondrocytes are not renewed, so this activity should be maintained long-term, if chondrocytes are edited [3,22].

The animals treated with LP+C+D showed increased IDUA activity in the synovial fluid, while the animals treated with LP+D and the untreated animals showed low levels of IDUA activity. Similar to the short term data, the enzyme was not increased in serum, liver, or kidney. These results indicate once again, that editing happened only in situ. As previously mentioned, the accumulation of GAGs causes hyperproliferation of synovial membrane cells, especially fibroblasts. Perhaps for this reason, enzyme activity was so high in synovial fluid [3]. In addition, it receives enzyme from the transfected cells of the joint [5].

Despite the low frequency of edited cells, enzyme activity in the joint tissue reached 2% of WT animals. Lysosomal disorders are known to benefit from low enzyme levels, although the exact proportion of editing needed to reach a clinical benefit in unknown and varies on many factors, such as the type of promoter used in the construct and the amount of enzyme secreted by the edited cell. Therefore, it was important to assess if the IDUA activity was sufficient to decrease specific GAG levels in the joint and the synovial fluid. The three injections significantly reduced the levels of GAGs but were not enough to completely normalize them. 

Liposomal delivery of CRISPR/Cas9 represents a significant step forward for the treatment of mucopolysaccharidosis type I (MPS I) when compared with previously reported viral-based gene delivery methods [9]. One of the main advantages of liposomal systems is their minimal risk of random genomic integration, in contrast to viral vectors, which are associated with a higher risk of insertional mutagenesis. This profile translates into an improved in vivo safety margin, a critical consideration for therapies intended for long-term or systemic use. In addition, liposomal formulations generally exhibit lower immunogenicity than viral vectors, which often elicit moderate to high immune responses that can limit therapeutic efficacy and preclude repeated administrations. The reduced immune activation associated with liposomal carriers allows for greater feasibility for re-dosing, an important advantage in chronic, multisystemic disorders such as MPS I. Liposomal systems also offer greater flexibility in cargo size capacity, enabling the delivery of diverse CRISPR/Cas9 formats, including plasmid DNA, mRNA, or ribonucleoprotein complexes, without the strict packaging constraints imposed by viral capsids [23]. 

Another key benefit of liposomal delivery is the ability to modulate tissue targeting through rational design of lipid composition, surface charge, and functionalization with targeting ligands. While viral methods rely largely on intrinsic vector tropism, liposomal systems can be engineered to optimize biodistribution and cellular uptake in specific tissues affected in MPS I. Collectively, these features contribute to a more favorable safety and efficacy profile in vivo [23]. Overall, liposomal CRISPR/Cas9 delivery combines the precision and long-term therapeutic potential of genome editing with a safer, nonviral platform. Preclinical studies indicate that this approach can overcome several limitations of viral gene therapy and traditional enzyme replacement therapy, supporting its promise as an advanced therapeutic strategy for MPS I.

Furthermore, the observed peak in IDUA activity at Day 7 followed by a substantial decline at Day 30 suggests that transgene expression, although initially robust, is not fully sustained over longer periods under the current experimental conditions. While the present study evaluated a three-monthly administration schedule based on translational feasibility and safety considerations, we acknowledge that more frequent dosing may enhance sustained enzymatic activity. Future studies should therefore investigate shorter dosing intervals, including weekly administrations, to determine whether repeated delivery improves durability of expression without increasing cytotoxicity or immunogenicity. Such optimization will be essential to define the most effective treatment schedule for long-term therapeutic benefit. Delivery of the editing system in other forms, such as mRNA, could also improve efficiency.

The low gene editing efficiency (0.007%) indicates the need for further optimization. Future strategies should focus on improving delivery efficiency through lipid formulation refinement, targeted delivery, and optimized dosing schedules. Enhancing HDR rates using DNA repair modulators, improved donor template design, or advanced editing platforms such as base or prime editing may also increase integration efficiency. Additionally, ex vivo editing approaches could allow for enrichment of corrected cells prior to administration. Importantly, in lysosomal storage disorders, even modest increases in editing efficiency may yield meaningful therapeutic benefit due to cross-correction, supporting continued optimization of this approach.

Key next steps toward clinical translation include improving editing efficiency and durability, conducting comprehensive safety and off-target analyses, evaluating biodistribution and long-term toxicity in relevant animal models, and establishing scalable GMP-compliant manufacturing. Demonstrating efficacy and safety in clinically relevant preclinical models will be essential before progressing to human trials.

Previous studies have shown that the delivery of ERT and gene therapy [5,24] to the joints of animals with MPS, aimed at treating joint problems, results in rapid synovial and joint IDUA activity and reduction of GAG levels without effects in non-target organs. Our results add important information regarding the safety and feasibility of such treatment for MPS patients, as well as other joint diseases.

## 5. Conclusions

This study investigated the potential of liposomes as nonviral delivery systems for the CRISPR/Cas9 gene-editing platform in both in vitro and in vivo settings, with a specific focus on treating the articular manifestations of MPS I. The findings demonstrated that cationic liposomes were capable of efficiently delivering CRISPR/Cas9 components to target tissues, resulting in a localized increase in IDUA enzymatic activity. This localized restoration of enzyme activity is particularly relevant for joint tissues, which are traditionally difficult to access and poorly responsive to conventional systemic therapies, including enzyme replacement therapy. Importantly, the use of liposomes enabled targeted delivery while maintaining a favorable safety profile, supporting their feasibility as nonviral vectors for genome editing. Overall, these results highlight the therapeutic promise of liposome-mediated CRISPR/Cas9 delivery for MPS I and potentially other genetic disorders that affect hard-to-reach tissues. This strategy represents a meaningful step forward in the development of advanced, tissue-targeted gene therapies with improved safety and translational potential.

## Figures and Tables

**Table 1:** Physicochemical properties of formulations and complexes.

Formulation	Mean Diameter (nm)	P.I.	ζ-Potential (mV)
LP	90.7 ± 3.5	0.26 ± 0.03	+32.4 ± 1.2
LP+D	117.5 ± 12.7	0.19 ± 0.04	+26.9 ± 2.1 *
LP+C+D	110.1 ± 2.5 *	0.16 ± 0.05	+27.2 ± 1.7 *

Results represent the mean ± standard deviation of three experiments. P.I.: polydispersity index; LP: Liposome; LP+D: liposome associated with IDUA donor plasmid; LP+C+D: liposome associated with CRISPR/Cas9 plasmid and IDUA donor plasmid for both in vivo and in vitro experiments. P.I.: polydispersity index. Statistical analysis was performed using one-way ANOVA and Tukey’s post hoc test, * *p* < 0.05 indicated difference from LP.

## Data Availability

The raw data supporting the conclusions of this article will be made available by the authors on request.

## Abbreviations

The following abbreviations are used in this manuscript:

CRISPR/Cas9	Clustered Regularly Interspaced Short Palindromic Repeats associated protein
DS	Dermatan sulfate
HS–OS	Heparan sulfate OS
HS–NS	Heparan sulfate NS
FLS	Fibroblast-like sinoviocyte
LF	Lipofectamine 3000^®^
LP	Liposome
LP+D	Liposome+Donor IDUA
LP+C+D	Liposome+CRISPR+Donor IDUA
M6-P	Mannose-6-fosfate
GAG	Glycosaminoglycan
IDUA	α-L-iduronidase
MPS I	Mucopolysaccharidosis type I
